# Extinction of Nicotine and Cocaine Seeking in Rats Reveals Novel, Unique and Time-Dependent Molecular Adaptations in the Medial Prefrontal Cortex

**DOI:** 10.1007/s10571-026-01667-0

**Published:** 2026-01-14

**Authors:** Caspar Muenstermann, Sarah J. Baracz, Eilish C. Heffernan, Nicholas C. Lister, Paul D. Waters, Kelly J. Clemens

**Affiliations:** 1 School of Psychology, University of New South Wales, UNSW, Sydney, NSW Australia; 2 School of Biotechnology & Biomolecular Sciences, UNSW, Sydney, Australia

**Keywords:** Addiction, Nicotine, Cocaine, RNA-Seq, ATAC-Seq, Intergenic, Incubation of craving

## Abstract

Nicotine dependence is characterized by high relapse rates compared to other addictive substances, yet the molecular mechanisms underlying relapse vulnerability during early abstinence remain poorly understood. Here we provide the first integrated transcriptomic and epigenomic profile of nicotine extinction in the medial prefrontal cortex (mPFC). Using RNA-seq and ATAC-seq at 1 and 6 days after nicotine or cocaine self-administration, we uncovered a dynamic and drug-specific molecular response. Nicotine was associated with minimal changes at day 1 but robust transcriptional and chromatin remodelling at day 6, possibly consistent with incubation of craving. Notably, we identified sustained upregulation of the dual specificity phosphatase *Dusp4* (first report in nicotine), implicating compensatory regulation of MAPK signalling in abstinence-related plasticity. Chromatin accessibility changes were enriched in intergenic regions containing FOS and JUND motifs, possibly indicative of enhancer-mediated transcriptional control rather than promoter remodelling. Together, these findings highlight nicotine-specific, time-dependent molecular adaptations in the mPFC and identify MAPK phosphatase signalling and enhancer activity as potential targets for relapse prevention during early abstinence.

**Supplementary Information:**

The online version contains supplementary material available at 10.1007/s10571-026-01667-0.

## Introduction

Cocaine is a highly reinforcing psychostimulant that robustly engages mesocorticolimbic circuits and produces enduring neuroplastic and transcriptional adaptations that drive compulsive drug seeking and relapse (Nestler 2012, 2014). Tobacco smoking is the leading cause of preventable disease worldwide, contributing to 8 million deaths per year (Reitsma et al., 2021). It is well established that abstinence from smoking requires inhibitory control to maintain abstinence even in the presence of cravings (Wray et al. 2013). This involves activation of the medial prefrontal cortex to exert inhibitory control over motor and reward pathways that ultimately trigger a resumption of drug seeking (Augur et al. 2016).

A growing body of research has now linked drug exposure with an array of epigenomic and transcriptomic changes that contribute to neuronal plasticity and function within the brain (Nestler 2012). Such approaches have signficantly increased our understanding of the biological mechanisms occurring in response to acute drug exposure including induction of plasticity-related immediate early genes, kinase/phosphatase signaling and modulation of downstream effectors such as CREB (Robison and Nestler 2011), however how addictive drugs like nicotine and cocaine lead to lasting changes that influence later drug seeking is less clear. Although cocaine is typically considered a stronger reinforcer that induces pronounced molecular and behavioral adaptations, nicotine paradoxically produces exceptionally high relapse rates despite comparatively weaker acute reinforcing effects (Palmatier et al. 2012; Carmody 1992). Understanding the molecular underpinnings of this dissociation is therefore critical for the development of effective therapeutic strategies to prevent relapse.

Gene expression changes and chromatin accessibility are crucial for neuroplasticity in response to addictive substances (Nestler 2014). Increasing evidence shows that multiple substances of abuse, prominently nicotine and cocaine, regulate chromatin accessibility at genes critical for memory and addiction leading to a range of neuroadaptations (e.g. synaptic plasticity and long-term potentiation; (Muenstermann and Clemens 2023; Pierce et al. 2018). While the persistence of epigenetic modifications beyond the cessation of drug intake has been demonstrated for several addictive drugs and linked to drug cravings and relapse (Cates et al. 2018; Li et al. 2015b; Nestler 2014), the persistence of epigenetic alterations beyond 24 h after nicotine or cocaine administration remains largely unexplored. Clarifying this process is important as epigenetic modifications may influence plasticity associated with the formation of new ‘no-drug’ memories in previously drug-paired contexts (Malvaez et al. 2010; Wang et al. 2010), particularly as extinction memories make use of the same plasticity targets as those impacted by nicotine (Ghitza et al. 2010).

The present study aimed to fill this gap by profiling gene expression and chromatin accessibility changes that occur across extinction of nicotine seeking when rats are drug free, potentially identifying changes in the mPFC that may underlie later susceptibility to relapse. Critically, a large body of preclinical work shows that the mPFC plays a causal role in relapse-like behaviours for both nicotine and cocaine: hyperactivity or aberrant ensemble activity in dorsal/prelimbic mPFC promotes cue-driven seeking and reinstatement, whereas infralimbic circuits support inhibitory control and extinction memory (Esaki et al. 2021; Huang et al. 2024; Izumi et al. 2021; Liu et al. 2024; Moorman and Aston-Jones 2023; Shin et al. 2016; Struik et al. 2019; Visser et al. 2022). Thus, transcriptional and epigenetic changes detected in the mPFC after nicotine or cocaine exposure are well positioned to reflect mechanisms that shift the balance between relapse-promoting and relapse-suppressing prefrontal outputs (Augur et al. 2016; Van den Oever et al. 2010). Extinction training in particular provides a valuable window into the neurobiological processes that emerge during early abstinence, when drug-associated memories are suppressed but not erased. Profiling molecular adaptations during extinction can therefore reveal the neuroplastic changes that maintain vulnerability to relapse, even in the absence of acute drug exposure.

Rats were trained to self-administer nicotine or cocaine followed by 1 or 6 days of extinction of drug self-administration to identify early and maintained transcriptomic and epigenomic regulation by drug exposure. The extinction time points for analysis were selected to mimic the periods of human drug abstinence that are most vulnerable to drug relapse (Westman et al. 1997). Transcriptomic and epigenomic changes caused by previous drug intake were assessed using bulk RNA and Assay for Transposase-Accessible Chromatin (ATAC) sequencing of the medial portion of the mPFC.

We hypothesized that extinction of nicotine seeking would produce distinct patterns of gene expression and chromatin accessibility changes in the mPFC compared to saline control, that the magnitude and scope of transcriptomic and epigenomic changes would differ with time since exposure, and that nicotine extinction would produce a unique molecular signature distinct from cocaine extinction. Due to the association between nicotine and chromatin modifications, we expected changes in chromatin accessibility to be associated with altered gene expression patterns, suggesting epigenetic regulation of transcription during nicotine extinction.

## Methods

### Animals

Thirty-two male Sprague Dawley rats (175–200 g; Australian Resources Centre, Perth, Australia) were housed, four per cage in a temperature and humidity-controlled room on a 12 h reverse dark/light cycle (light on at 19:00 h, off at 07:00 h). Rats were handled and underwent surgery for intravenous catherization as described previously (Macnamara et al. 2016).

All procedures were approved by the Animal Care and Ethics Committee of the University of New South Wales (Project 21/93B) and were conducted in accordance with the Australian Code for the Care and Use of Animals for Scientific Purposes (8th ed.).

## Intravenous Self-administration

**Fig. 1:**
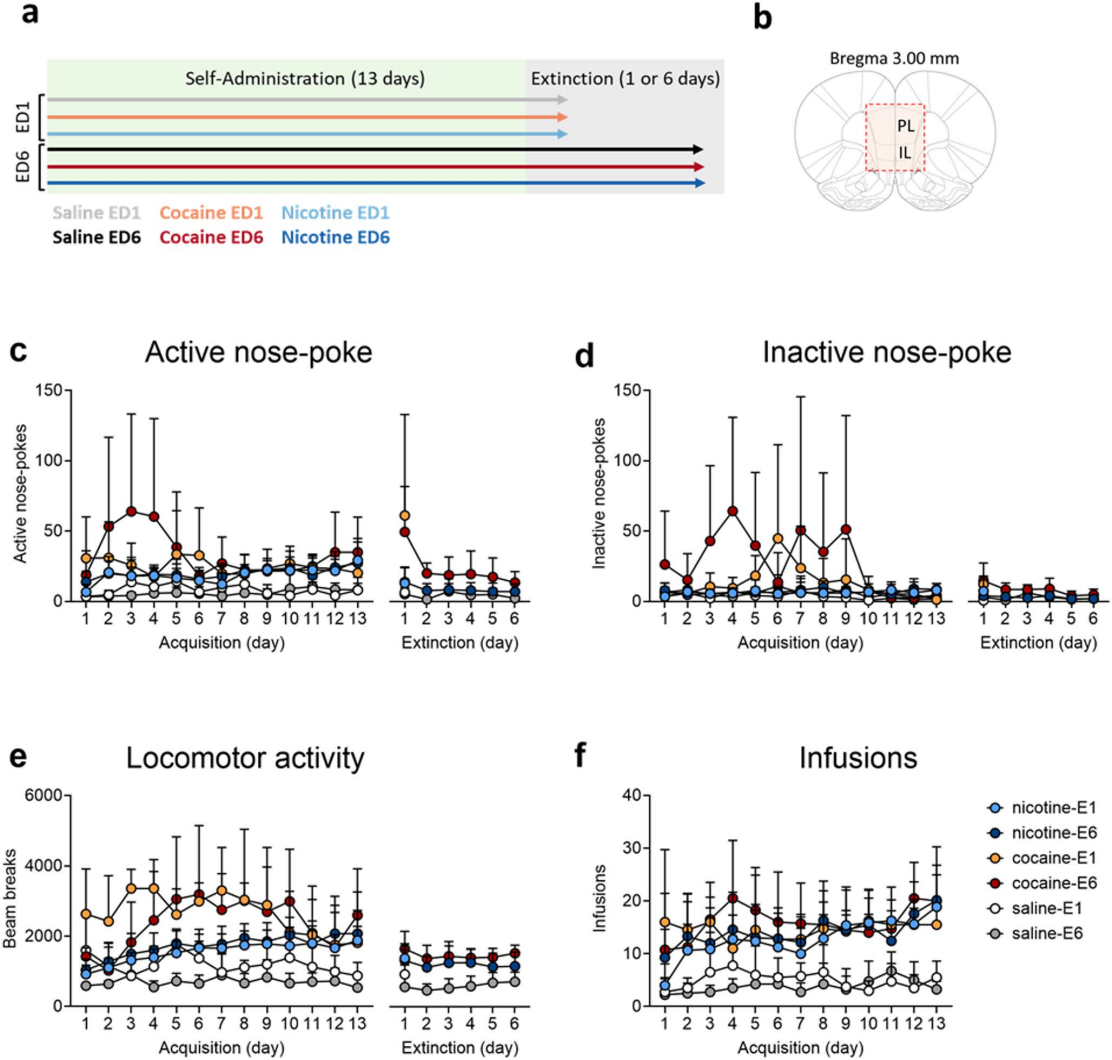
Nicotine and cocaine support intravenous self-administration. **a** Overall procedure. **b** brain regions dissected for analysis. Self-administration acquisition and extinction behavioural data **c** active nose-pokes, **d** inactive nose-pokes, **e** locomotor activity and **e** infusions. Data points represent group mean *±* standard deviation. *IL* infralimbic, *PL* prelimbic, *ED1* extinction day 1, *ED6* extinction day 6. *n* = 4 (saline and cocaine) to 8 (nicotine) rats per group to generate tissue for sequencing

Procedures and equipment were consistent with that used previously (Macnamara et al. 2016). Rats were assigned to one of three treatment conditions and trained to self-administer cocaine (*n* = 8; 750 µg/kg/50 µL infusion), saline (*n* = 8; 100 µL infusion) or nicotine (*n* = 16; 30 µg free base/kg/100 µL infusion; Fig. 1a). Sample sizes were selected based on prior RNA-seq studies showing that 4–6 biological replicates per condition provide sufficient power (> 80%) to detect ≥ 1.5-fold expression changes at FDR < 0.05, given typical dispersion estimates in mammalian brain tissue (e.g., (Conesa et al. 2016; Schurch et al. 2016). More rats were assigned to the nicotine condition due to greater variability in responding compared to cocaine (Clemens et al. 2010). Rats were trained to nose-poke for an infusion and paired visual cue for 1 h per day across 13 days followed by 1 or 6 extinction sessions (house-light on, no outcome). Rats were counterbalanced across extinction day 1 and day 6 conditions to ensure comparable histories of drug exposure. Thirty mins after the extinction session rats were euthanized with intravenous pentobarbitone sodium (325 mg/ml/rat) and brains extracted. The ventromedial prefrontal cortices incorporating both the infralimbic and prelimbic cortex (approx. 2.52–5.16 mm bregma) were dissected and stored as separate hemispheres (Fig. 1b).

## RNA Sequencing

One mPFC hemisphere from each rat underwent RNA extracted using the TRIzol method according to manufacturer instructions (Invitrogen), followed by purification using a RNeasy mini spin column (Qiagen, NRW, Germany). All RNA samples were quality assessed, ribosome depleted and libraries prepared using the Illumina TruSeq stranded RNA library kit (Illumina, CA, USA). Library preparation was performed on the Illumina NovaSeq 6000 XP platform by the Western Sydney University Next-Generation Sequencing Service (Richmond, Australia). Sequencing was 100 bp paired end and approximately 25 million reads were generated per sample.

Outputs underwent quality control (Andrews 2010) and trimmed (Bolger et al. 2014) then mapped to the Rnor6 reference genome using Hisat2 (Kim et al. 2019). Count matrices were generated using the HT-seq (Anders et al. 2015). Differential gene expression analysis was performed using edgeR (Robinson et al. 2010). Genes were considered as significantly regulated at an initial False Discovery Rate (FDR) of < 0.2 using glm tests. A higher FDR of 0.2 was included as low-level gene regulation was expected in the absence of acute drug exposure and the exploratory nature of the study. Data relating to a more stringent FDR of < 0.05 is also indicated. Regulated biological functions were assessed by using differentially expressed genes (DEGs) in an Overrepresentation analysis (ORA) using clusterProfiler (Wu et al. 2021) and corrected using a Benjamin-Hochberg correction with a q-value cutoff of 0.05. To determine overlap in patterns of gene regulation we used a Rank-Rank Hypergeometric overlap (RRHO) analysis using RRHO2 (Cahill et al. 2018). Transcription factor foot printing was performed using the Signac package (Stuart et al. 2021), and enriched motifs in DEGs over background were determined using the FindMotifs function with a p-value cutoff of 0.05.

## ATAC Sequencing

Nuclear extraction of the alternate mPFC hemisphere was performed using the Omni-ATAC protocol for frozen tissues (Corces et al. 2017). Fifty-thousand nuclei were aliquoted and incubated in Illumina Transposase (Illumina, CA, USA) then purified using Zymo DNA clean and concentrator kit (Zymo, CA, USA). Library quantification was performed using the KAPA library quantitation kit (Roche, BS, Switzerland). Sequencing (120 million 150 bp paired end reads) was performed on the Illumina Novaseq-6000 instrument by the Ramaciotti Centre for Genomics (UNSW Sydney, Australia). Reads were mapped to the rat genome using bowtie2 (rn7) (Langmead and Salzberg 2012). Peak files for each sample were merged into a consensus peak set using Bedtools (Quinlan and Hall 2010). Counts in each peak for each sample were obtained using the featureCounts function of the Rsubreads package (Liao et al. 2014). Differential accessibility analysis of peak counts was performed using edgeR (Robinson et al. 2010). Significant differentially accessible regions (DARs) were considered at FDR < 0.2 using glm tests due to the exploratory nature of the study. Significant peaks were annotated to their genomic location using ChipSeeker (Wang et al. 2022). DAR were annotated to nearby gene promoters within 1 kb using the detailRanges functions in the csaw package (Lun and Smyth 2016) using gene promoters identified in the TxDb.Rnorvegicus.UCSC.rn7.refGene database. DARs corresponding to gene promoter regions were used for ORA analysis to determine molecular functions associated with chromatin regulation using clusterProfiler (Wu et al. 2021). ORA was corrected using a Benjamin-Hochberg correction with a q-value cutoff of 0.05 to determine significance. A Rank-Rank Hypergeometric overlap (RRHO) analysis on the entire genome was performed using RRHO2 (Cahill et al. 2018) was performed and results ranked based on a differential expression score obtained based on the rule: ∗−10(−). Transcription factor foot printing was performed using the Signac package (Stuart et al. 2021), and enriched motifs in DARs over background were determined using the FindMotifs function with a p-value cutoff of 0.05.

## Comparative Analysis

Comparative analysis was performed using previously created gene and peak count matrices in RStudio where datasets were filtered down to genes that overlap with a present gene promoter chromatin peak in the ATAC-seq dataset. Both peaks and genes were ranked based on the rule: ∗−10(−) and compared using an RRHO analysis of the entire genome using the RRHO2 package (Cahill et al. 2018).

A summary of FDR thresholds is provided in Table S1.

## Results

### Nicotine and Cocaine Support Robust Intravenous Self-administration, and this is at a Higher Rate for Cocaine

Behavioral data of rats generating tissue for sequencing are shown in Fig. 1a–b, illustrating that although the rate of acquisition differed between drugs (day by drug interaction: F(24,300) = 2.043, *p* < 0.01), response rates were stable across the final five days of training where both nicotine- and cocaine-trained rats made significantly more nose-pokes than saline controls (F(2,25) = 9.578. *p* < 0.01, Tukey’s post-hoc Sal vs. Coc *p* < 0.01; Sal vs. Nic *p* < 0.01), but nicotine and cocaine were not different from each other (*p* = 0.859). Cocaine and nicotine rats took significantly more infusions than saline controls across training (F(2,25) = 17.705, *p* < 0.001, post-hoc *p* < 0.001) and were significantly more active (F(2,25) = 14.443, *p* < 0.001, post-hoc *p* < 0.01). Rats subsequently assigned to ED1 or ED6 responded to an equivalent level (Fs < 1.0).

On day 1 of extinction, cocaine rats made more nose-pokes than nicotine or saline, and the latter did not differ from each other (F(2,25) = 8.388, *p* < 0.01; saline vs. cocaine *p* = 0.003; nicotine vs. cocaine *p* = 0.005). The same held after 6 days of extinction (F(2,12) = 6.277, *p* < 0.05; saline vs. cocaine *p* = 0.018; nicotine vs. cocaine *p* = 0.028), where all rats (except one cocaine rat) achieved a > 60% reduction in responding compared to an average of the last 3 days of self-administration. This pattern of self-administration and extinction is consistent with past studies in this area (Clemens et al. 2010; Macnamara et al. 2016) and was not associated with somatic signs of withdrawal.

### Nicotine and Cocaine Lead to a Dynamic and Drug-Specific Pattern of Differentially Expressed Genes

**Fig. 2:**
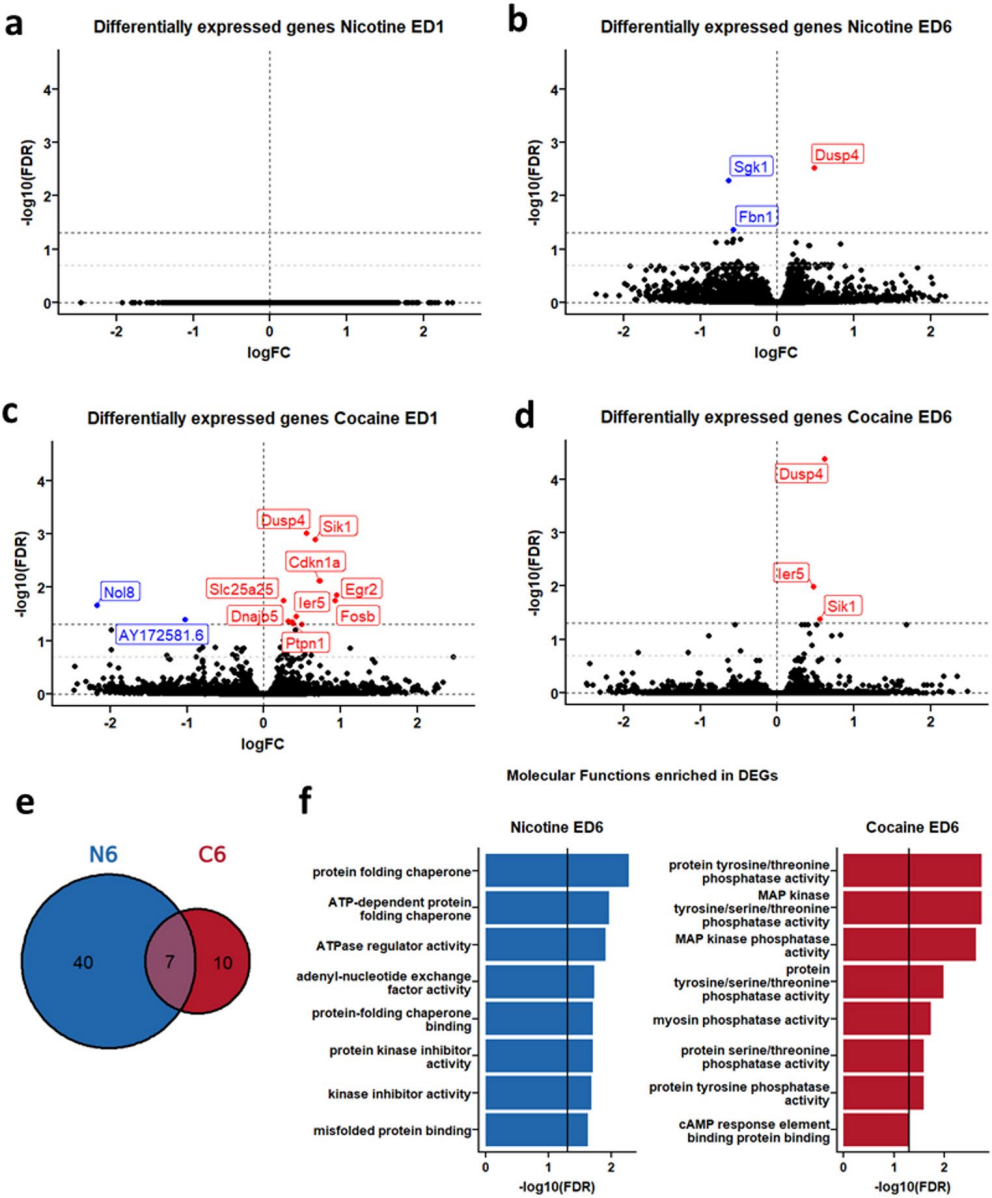
Nicotine and cocaine lead to a dynamic and drug-specific pattern of differentially expressed genes. **a–d** Volcano plots of transcriptional changes in the medial prefrontal cortex (mPFC) following extinction of nicotine or cocaine self-administration. Genes at false discovery rate (FDR) < 0.05 are annotated. Red labels indicate genes with increased expression, blue labels indicate genes with decreased expression. **e** Venn diagram showing overlapping differentially expressed genes between extinction day 6 of nicotine and cocaine groups. **f** Significantly enriched molecular function ontology terms at extinction day 6 of nicotine and cocaine groups. *FC* fold change, *ED* extinction day, *DEG* differentially expressed gene. *n* = 4 (saline and cocaine) to 8 (nicotine) rats per group

In the absence of acute drug administration, low absolute numbers of differentially expressed genes were observed compared to saline controls. This was particularly true at extinction day 1 with no DEGs in the nicotine group (Fig. 2a) and 54 DEGs in the cocaine group (19 DEGs at FDR < 0.05; Fig. 2b; Table S2). The pattern at extinction day 6 differed substantially, where 47 DEGs were now observed in the nicotine group (4 DEGs at FDR < 0.05; Fig. 2c; Table S3), and 17 DEGs in the cocaine group (5 DEGs at FDR < 0.05; Fig. 2d; Table S4). Similarities were observed in gene regulation between nicotine and cocaine groups, with 7 DEGs being present in both groups at extinction day 6, although the majority (40) of day 6 DEGs were unique to nicotine (Fig. 2e; Table S5). There were no DEGs between saline extinction day 1 and 6.

To infer function of differentially expressed genes, ORA revealed no clear enrichment at extinction day 1, however there were 11 significantly enriched ontology terms at extinction day 6 of nicotine, and 13 significantly enriched ontology terms at extinction day 6 of cocaine (Fig. 2f; Table S6). As observed with differentially expressed genes, enriched molecular functions overlapped between nicotine and cocaine, showing a shared regulation of protein kinase inhibitor activity, related in particular to MAP kinase phosphatases *DUSP4* and *DUSP6*. Correlational analysis indicated that *DUSP4*,* Arc*,* Dnajb5* and *Egr2* show a significant correlation with nicotine intake across the last 3 days of nicotine self-administration indicating that gene expression may be related to behavioural output (Fig S1).

**Fig. 3:**
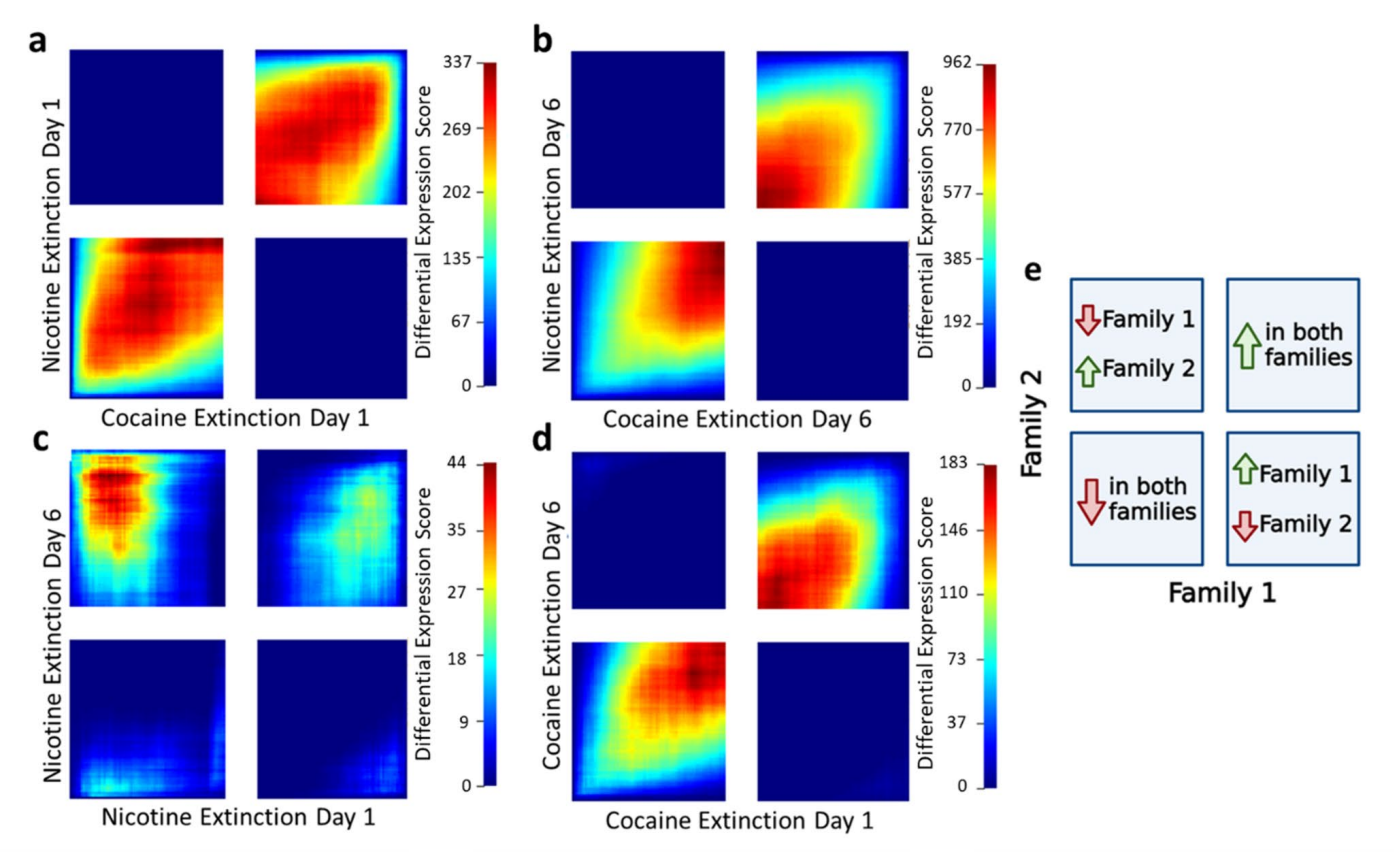
Rank-Rank Hypergeometric Overlap (RRHO) analyses confirming similar transcriptomic regulation between nicotine and cocaine. RRHO compares lists of genes ranked by logFc*-log10(pval) between **a** extinction day 1(ED1) of cocaine and nicotine, **b** extinction day 6 (ED6) of cocaine and nicotine, **c** ED1 and ED6 within nicotine and **d** ED1 and ED6 within cocaine. **e** Legend for RRHO analyses where quadrants indicate the direction of shared gene regulation between the two datasets: top-right = genes up-regulated in both; bottom-left = genes down-regulated in both; top-left and bottom-right represent opposing regulation (e.g., up in one dataset and down in the other). Family 1 refers to the x-axis dataset and Family 2 refers to the y-axis data set as indicated on figures a-d. *n* = 4 (saline and cocaine) to 8 (nicotine) rats per group

RRHO analysis confirmed similar transcriptomic regulation between nicotine and cocaine where genes up or downregulated by one often correlated with the other (Fig. 3a, b) when compared to saline baseline (saline day 1 and 6 combined). Further signal was observed in the upper right and lower left quadrants when comparing extinction day 1 of cocaine to extinction day 6 of cocaine (Fig. 3d), indicating that a history of cocaine self-administration resulted in long lasting transcriptomic changes. The same was however not true for nicotine, with little signal observed in any quadrant in the comparison of transcriptome at extinction day 1 and 6 of nicotine (Fig. 3c).

### Changes in Chromatin Accessibility are Greatest After 6 days of Extinction for Both Nicotine and Cocaine

**Fig. 4:**
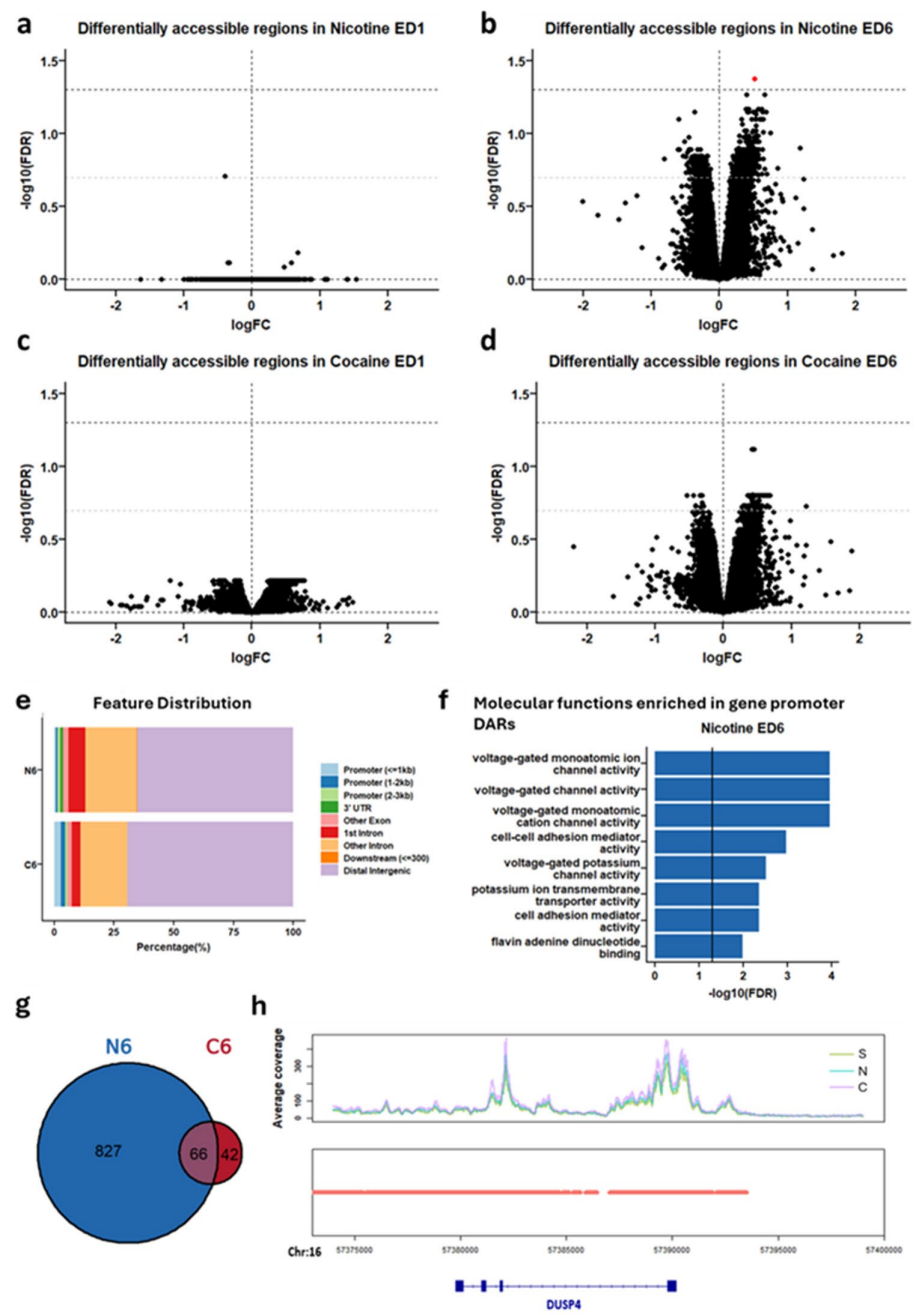
Six days of extinction of nicotine and cocaine self-administration is associated with change of chromatin accessibility in the prefrontal cortex. **a–d** Volcano plots showing differentially accessible regions at extinction day 1 (ED1) or extinction day 6 (ED6) of nicotine and cocaine self-administration. **e** Breakdown of chromatin accessibility on day 6 of extinction from nicotine seeking as functional Annotation of differentially accessible chromatin peaks **f** Significantly regulated molecular function gene ontology terms of differentially accessible gene promoters at extinction day 6 of nicotine. **g** Venn diagram showing the overlap of differentially accessible chromatin peaks at extinction day 6 between nicotine and cocaine groups **h** Gene track plot showing the accessibility of the Dusp4 gene across drug conditions. *S *saline, *N *nicotine, *C *cocaine. *DAR *differentially accessible region, *ED *extinction day, *N6 *nicotine extinction day 6, *C6* cocaine extinction day 6. *n* = 4 (saline and cocaine) to 8 (nicotine) rats per group

Similar to patterns observed in transcriptional data, little differential chromatin accessibility was observed at extinction day 1, with a single differentially accessible region for the nicotine group and none for the cocaine group at FDR < 0.2 (Fig. 4a, c). Markedly greater differential chromatin accessibility was observed at extinction day 6 with 893 differentially accessible regions for nicotine and 108 differentially accessible regions for cocaine (Fig. 4b, d).

Chromatin change for both drugs at extinction day 6 showed more accessible chromatin and annotated to mostly intergenic and intronic regions of the genome. Only 1–4% of differentially accessible chromatin regions were located near annotated gene promoters (Fig. 4e) and none were located near a gene promoter that was identified as a DEG in the RNA sequencing data.

Differentially accessible chromatin regions that +/- 1 kb of a gene promoter show 14 overrepresented molecular functions at extinction day 6 of nicotine, indicating altered accessibility at genes important for voltage gated ion channel activity (Fig. 4f; Table S8). At extinction day 6, 66 differentially accessible regions were shared between nicotine- and cocaine-exposed groups (Fig. 4g; Table S7). In contrast, examination of the *Dusp4* locus (Fig. 4h) revealed uniformly high chromatin accessibility across all conditions, suggesting that the nicotine-associated upregulation of *Dusp4* expression was not linked to changes in local promoter accessibility.

### Nicotine and Cocaine Produce a Similar Pattern of Transcription Factor Motif Enrichment

Transcription factor (TF) motif enrichment analysis was performed on DARs at extinction day 6 of nicotine and cocaine. On extinction day 6 of nicotine, 30 enriched TF motifs were identified, while 27 were identified at extinction day 6 of cocaine (Table S9). We identified 27 shared transcription factor binding motifs between nicotine and cocaine conditions, suggesting these differentially accessible regions may be targets of the same transcription factors and thus share common biological regulatory mechanisms. Several of the observed TF motifs are known targets of drug exposure including JUND and FOS (Fig. 4a). No overlapping enriched TF motifs were identified in the RNA-sequencing datasets, indicating differentially expressed genes may not be regulated by the same TFs (Table S10).

**Fig. 5:**
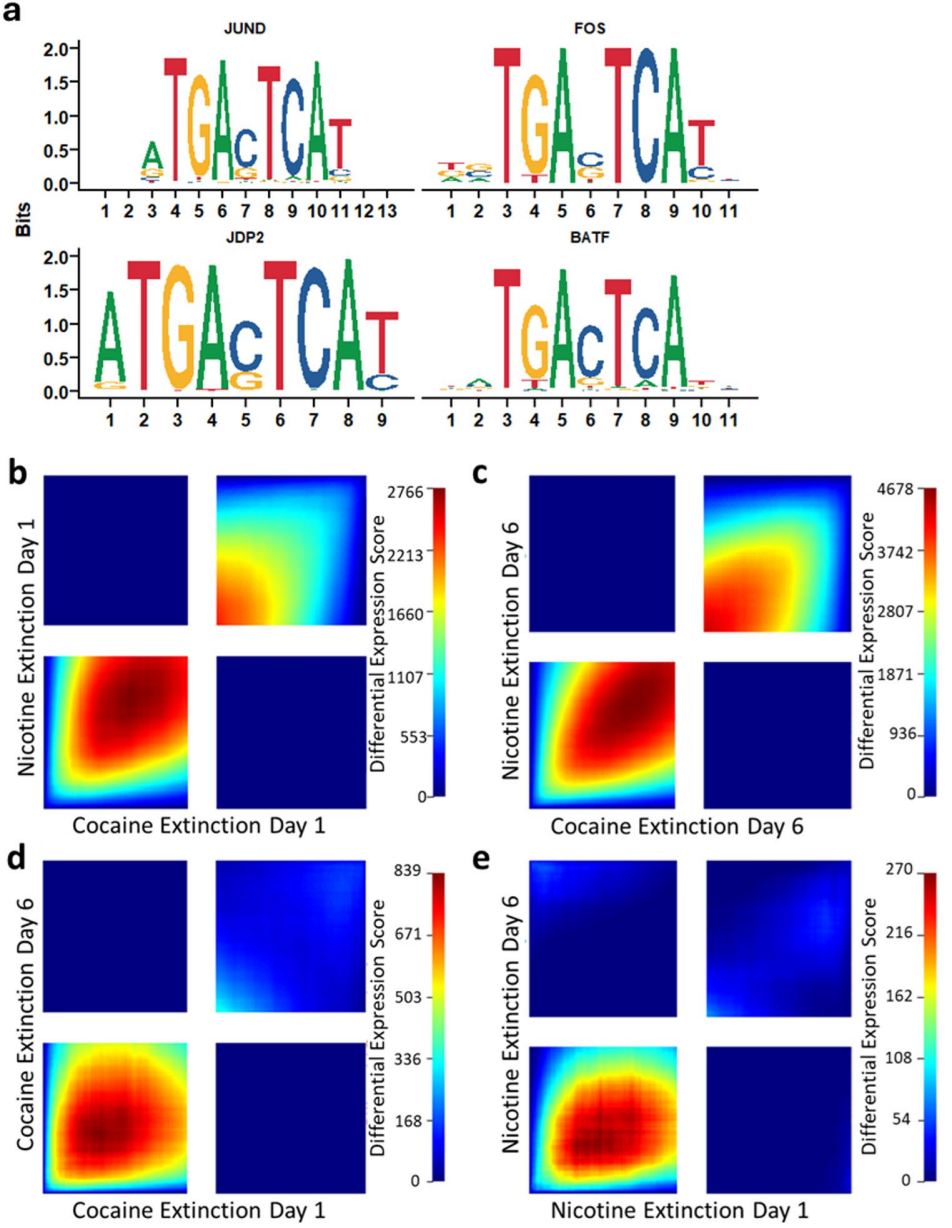
Nicotine and cocaine produce a similar pattern of transcription factor motif enrichment. **a** Four transcription factor motifs that are enriched following extinction day 6 of both nicotine and cocaine. Rank-Rank hypergeometric overlay analyses comparing lists of ATAC-peaks ranked by logFc*-log10(pval) between **b** extinction day 1 of cocaine and nicotine, **c** extinction day 6 of cocaine and nicotine, **d** extinction day 1 and extinction day 6 within cocaine **e** and extinction day 1 and extinction day 6 within nicotine. n = 4 (saline and cocaine) to 8 (nicotine) rats per group

Similar to patterns observed in transcriptomic data, RRHO analysis revealed similar chromatin accessibility at both extinction day 1 and 6 (Fig. 5b, c), indicating regulation of chromatin accessibility was largely consistent between nicotine and cocaine. Concordant decreases in chromatin accessibility were observed between extinction day 1 and 6 in both drug groups, indicating that regions of decreased chromatin accessibility may be maintained from early to late extinction (Fig. 5d, e).

### Changes in Gene Expression were not Associated with Changes in Chromatin Accessibility

**Fig. 6:**
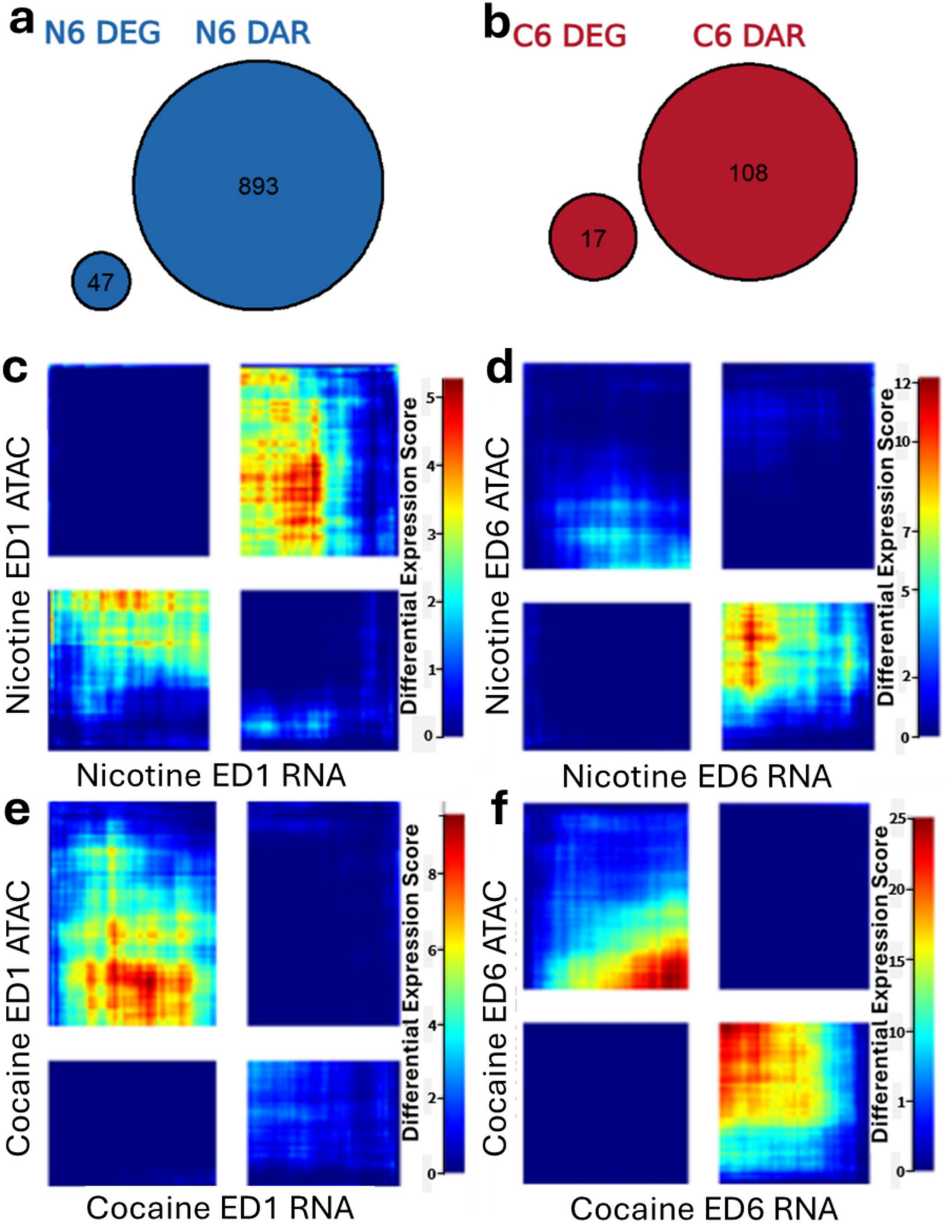
Changes in gene expression were not associated with changes in chromatin accessibility. Venn diagrams showing no overlap between differentially accessible regions identified at **a** extinction day 6 of nicotine and **b** extinction day 6 of cocaine and differentially expressed genes. **c**–**f** Rank-rank hypergeometric overlap analyses comparing chromatin accessibility of ATAC peaks in promoter regions and corresponding gene expression at each extinction day of nicotine and cocaine groups. *DAR* differentially accessible region, *DEG *differentially expressed gene, *ED *extinction day, *N6 *nicotine extinction day 6, *C6 *cocaine extinction day 6. *n* = 4 (saline and cocaine) to 8 (nicotine) rats per group

Little overlap was observed between differential gene expression and chromatin accessibility. No differentially accessible gene promoters were observed for differentially expressed genes, indicating that changes in gene expression were not associated with changes in chromatin accessibility (Fig. 6a, b). Further RRHO analysis show generally low signal, indicating that regions of unchanged chromatin accessibility generally showed high gene expression (Fig. 6c–f).

## Discussion

Our results highlight several distinct and time-dependent molecular processes that may contribute to the persistence of nicotine-seeking across time. We identified a unique pattern of DEGs in rats that had self-administered nicotine, with none detected on day 1 of extinction, versus 47 detected 6 days later. In contrast, DEGs decreased from 54 to 17 for rats previously self-administering cocaine. Altered gene expression was paralleled by an increase in chromatin accessibility from day 1 to day 6 of extinction for both cocaine and nicotine, although this did not overlap with the location of DEGs, but rather highlighted increased chromatin accessibility in intergenic and intronic regions of the genome. GO-term analysis revealed distinct drug-specific pathways of enrichment, where nicotine was associated with enriched protein folding and protein kinase activity, whereas cocaine samples showed enrichment of protein tyrosine/threonine phosphatase activity. Together these data suggest the presence of multiple drug-induced molecular changes that persist in the absence of the drug itself, and in particular unique patterns of molecular changes associated with nicotine versus cocaine.

Overall, molecular changes in the mPFC following nicotine were amplified with time since last exposure. This pattern of expression cannot be due to behavioural changes, as behavioural output (e.g. activity or nose-pokes) decreased over this period and gene expression in saline controls remained consistent. An increase in gene expression across abstinence is consistent the incubation of craving phenomena, previously described for both cocaine and nicotine (Pickens et al. 2011). Here, the extent to which animals’ relapse to drug seeking increases with time since last drug exposure (Li, Caprioli, et al., 2015), a pattern of behaviour that has been linked to a progressive increase in the expression of plasticity-related genes (Cates et al. 2018; Kuntz-Melcavage et al. 2009). Although the extent of abstinence in the current study was relatively short, this is consistent with incubation of craving following nicotine (Bedi et al. 2011) which may decline after as few as 7 days (Markou et al. 2018), and may reflect the comparatively fewer changes observed in cocaine, where incubation of craving has been observed for as long as 60 days with cocaine (Lu et al. 2004). Alternatively, changes in mPFC gene expression may be reflective of altered regulation of prefrontal cortex plasticity over time, and resultant impacts on inhibitory control over drug seeking (Van den Oever et al. 2010). Verifying the link between craving and gene expression will be an important next step for this research.

### Gene Expression: Nicotine Versus Cocaine

Differences between nicotine and cocaine were further reflected in the biological functions associated with these DEGs. GO-term analysis following nicotine revealed specific enrichment of genes associated with protein folding. This may reflect a response to nicotine-induced cellular stress, where increased expression of protein folding chaperones occurs as a compensatory mechanism to maintain homeostatic processes (Voisine et al. 2010), particularly in areas rich in dopamine signalling (Danilova et al. 2019). This finding contrasted with enrichment pathways following cocaine, that primarily identified phosphorylation pathways linked to plasticity, a pathway known to be altered following cocaine exposure (Besnard et al. 2011).

The RNA-seq data revealed significant change of expression of two dual specificity phosphatases, *Dusp4* and *Dusp6* at extinction day 6 of nicotine and cocaine self-administration. These proteins are key regulators of the MAPK signalling cascade, acting to decrease activity at downstream targets in the MAPK-CREB pathways via Erk1, Erk2, and c-Jun (Sun et al. 2016). This is the first indication of changes in *Dusp4* gene expression following nicotine exposure in rats, although there is extensive evidence of increased activation in this pathway following exposure to other addictive substances. For example, *Dusp6* is upregulated in multiple brain regions following acute methamphetamine exposure (Ujike et al. 2002), while *Dusp1*, *Dusp5* and *Dusp14* are upregulated following MDMA exposure (Marie-Claire et al. 2008).

MAPK signalling is known to mediate the rewarding and addictive properties of substances of abuse in general (Miller and Marshall 2005) and has been extensively implicated in cocaine, opioid, and methamphetamine craving (Cates et al. 2018; Li et al. 2008; Lu et al. 2005). Acute nicotine administration induces phosphorylated MAPK via calcium influx through nAChR binding (Utsugisawa et al. 2002). A novel finding here was the identification of MAPK enrichment increasing after 6 days of extinction training. This may be reflective of compensatory feedback loops engaged in response to chronic nicotine exposure and suggests that changes in *Dusp* regulation could be an indirect homeostatic response that contributes to plasticity over time.

Although the present findings are descriptive, the molecular pathways identified have well-established roles in extinction and relapse-related circuitry. Extinction training is widely characterized as a form of new learning rather than passive forgetting, and the molecular signatures we identify are consistent with activity-dependent synaptic plasticity that supports extinction memory formation. Several of the regulated genes and enriched motifs identified here (for example Arc, Egr2, and enrichment of FOS/JUND motifs) classically mark neuronal activity and synaptic remodelling, and the DUSP–MAPK changes we observe likely influences CREB phosphorylation and downstream transcriptional programmes that support long-term potentiation/depotentiation and memory consolidation (Miller and Marshall 2005; Augur et al. 2016). Thus, rather than being exclusively markers of drug exposure per se, the transcription factors and signalling cascades we detected may reflect processes engaged during extinction learning (consolidation or reconsolidation of ‘no-drug’ memories) and/or homeostatic responses that alter the ease with which extinction memories are formed or maintained. Given the prominence of CREB/MAPK in both memory and addiction literature, targeted manipulations (e.g., timed modulation of DUSP activity or CREB signalling during extinction) would help determine whether the identified pathways facilitate extinction learning (thereby protecting against relapse) or conversely weaken extinction consolidation and thereby increase relapse vulnerability.

### Chromatin Accessibility

Surprisingly, differentially accessible chromatin regions did not directly overlap with differentially expressed genes. Gene promoter accessibility is generally viewed as a primary regulator of gene expression (Orphanides and Reinberg 2002), with more accessible chromatin being associated with higher gene expression. However, our existing data somewhat contradicts this, raising several possibilities. First, promoter regions typically maintain high baseline accessibility across the genome and changes in gene expression may not necessarily be reflected by or require additional changes in promoter accessibility (Chereji et al. 2019), i.e. promoter regions may be ‘primed’(Mews et al. 2024). Second, the temporal dynamics of gene expression and transcriptional regulation may be misaligned (Voss and Hager 2014). Finally, there may be indirect regulatory elements, including long-range chromatin interactions and noncoding RNAs as mediators of extinction-induced transcriptional changes that could play a role (Kim et al. 2019; Statello et al. 2021).

Consistent with this last point, the vast majority of chromatin regulation in the present dataset occurred at previously unannotated intergenic regions. This highlights the potential for these regions to act as either enhancer regions of existing genes, or transcription sites for noncoding RNAs (Statello et al. 2021). In support of this conclusion, our motif analysis showed enriched TF motifs for drug associated TFs *FOS* and *JUND* in DARs (Teague and Nestler 2022). Transcription factors are known to regulate transcription by altering the 3-dimensional structure of chromatin, indicating that the differentially accessible chromatin regions may be enhancers regions that alter gene expression via chromatin looping (Kim et al. 2019). This interpretation is consistent with a growing literature consensus that many noncoding regions of DNA have crucial biological functions, regulating gene expression, gene splicing or chromatin structure (Isoda et al. 2017; Tripathi et al. 2012). Future investigation should highlight the importance of differentially accessible noncoding chromatin regions in transcriptional regulation and subsequent drug response.

## Limitations

Several limitations warrant consideration. First, without home-cage controls collected at matching time-points, we cannot fully dissociate molecular signatures of extinction learning from those of acute behavioral activation. However, saline controls exhibited minimal transcriptional or chromatin change, suggesting that the effects observed are drug-history dependent. Second, the exploratory FDR < 0.2 threshold was chosen to detect subtle molecular signals expected during drug-free extinction. Importantly, convergent findings across RNA-seq, ATAC-seq, and motif analyses, and consistency of top-ranked genes under a stricter FDR < 0.05, support the robustness of our interpretations.

A further limitation of the present sequencing datasets was the use of prefrontal cortex homogenate from male rats. Using a homogenate, the information around cell-type and subregion differences is lost, particularly given the separate inputs and projections to the infralimbic and prelimbic regions, although recent evidence suggests that both areas are likely to contribute to extinction and drug seeking (Moorman and Aston-Jones 2023). This does not dimmish the novelty of the global changes observed and rather suggests that the detected changes are likely robust. While our data reveals important insights, these findings may not generalize to females. This is supported by studies showing distinct pathways regulated during cocaine withdrawal between sexes in the nucleus accumbens (LaRese et al. 2019), and minimal overlap in differentially expressed genes in male and female mouse brains following acute ethanol exposure (Hitzemann et al. 2022). Furthermore, overexpression of *Dusp4* in mice results in sex-specific cognitive rescue in a mouse model of Alzheimer’s disease (Pan et al. 2022), highlighting the need to expand our approach across sexes.

## Conclusions and Future Directions

This study provides the first integrated transcriptomic and epigenomic profile of nicotine extinction and highlights several novel findings. We show for the first time that Dusp4 is upregulated in the mPFC following nicotine self-administration, alongside delayed *Dusp6* induction, consistent with compensatory feedback regulation of MAPK signaling. We also demonstrate that chromatin remodelling occurs predominantly in intergenic regions enriched for drug-associated transcription factor motifs (e.g., FOS, JUND), implicating enhancer-mediated regulation in nicotine-related plasticity. Finally, the dissociation between differentially expressed genes and chromatin accessibility challenges canonical assumptions about promoter regulation and identifies alternative regulatory layers driving abstinence-induced molecular change. Together, these findings identify distinct and time-dependent molecular processes that may contribute to incubation of nicotine craving and highlight MAPK phosphatase signalling and enhancer activity as promising therapeutic targets for relapse prevention.

In summary, our findings provide new insight into the molecular adaptations that emerge within the medial prefrontal cortex during early abstinence from nicotine. Although the study did not include direct measures of relapse or functional manipulations of identified targets, the observed regulation of MAPK phosphatase signalling and enhancer-linked chromatin remodelling points to mechanisms that may shape the persistence of nicotine seeking and the vulnerability to relapse. These extinction-phase molecular changes highlight that abstinence itself is an active period of neurobiological plasticity, rather than a passive return to baseline. The identification of DUSP-related regulation in particular suggests a compensatory modulation of MAPK–CREB signalling that may influence prefrontal control over drug-seeking circuits. Future studies combining transcriptomic and cell type–specific manipulations during extinction and reinstatement will be critical to determine whether these pathways actively govern relapse propensity. By establishing a foundational molecular map of nicotine extinction, the present study provides a platform for hypothesis-driven functional testing and for developing strategies aimed at strengthening inhibitory control during abstinence to prevent relapse.

## Supplementary Information

Below is the link to the electronic supplementary material.

Supplementary Material 1

Supplementary Material 2

Supplementary Material 3

Supplementary Material 4

Supplementary Material 5

Supplementary Material 6

Supplementary Material 7

## Data Availability

The datasets generated during the current study are not publicly available but are available from the corresponding author upon request.
