# Supplementary material for: Extinction of Nicotine and Cocaine Seeking in Rats Reveals Novel, Unique and Time-Dependent Molecular Adaptations in the Medial Prefrontal Cortex
Source: Cell Mol Neurobiol. 2026 Jan 14;46:29. doi: 10.1007/s10571-026-01667-0 (PMC12876482; doi:10.1007/s10571-026-01667-0)

**Supplementary Materials**

*Extinction of Nicotine and Cocaine Seeking in Rats Reveals Novel, Unique and Time-Dependent Molecular Adaptations in the Medial Prefrontal Cortex*

**Supplementary Table 1.** False discovery rate thresholds for the different data sets.

| **Analysis** | **Threshold** |
| --- | --- |
| DEGs / DARs (discovery) | FDR < 0.2 |
| DEGs (high-confidence) | FDR < 0.05 |
| GO-term enrichment | FDR < 0.05 |
| TF motif enrichment | FDR < 0.05 |

**Supplementary Table 2.** Differentially Expressed Genes following RNA-Seq: Saline Day 1 vs Cocaine Day 1 (FDR < 0.05; FDR <0.2)

|  | ENSEMBLID | Symbol | logFC | logCPM | LR | PValue | FDR |
| --- | --- | --- | --- | --- | --- | --- | --- |
| 28053 | ENSRNOG00000011921 | Dusp4 | 0.55988 | 5.079757 | 29.60261 | 5.30E-08 | 0.000972 |
| 31395 | ENSRNOG00000001189 | Sik1 | 0.672854 | 4.669227 | 27.72981 | 1.39E-07 | 0.001278 |
| 8036 | ENSRNOG00000059639 | AABR07059882.1 | -3.3854 | -2.71263 | 24.07585 | 9.26E-07 | 0.005658 |
| 14441 | ENSRNOG00000052357 | NA | 0.729605 | 2.979341 | 22.89394 | 1.71E-06 | 0.007362 |
| 31571 | ENSRNOG00000000521 | Cdkn1a | 0.719007 | 3.077199 | 22.58689 | 2.01E-06 | 0.007362 |
| 31330 | ENSRNOG00000000640 | Egr2 | 0.944009 | 4.823438 | 21.0312 | 4.52E-06 | 0.013801 |
| 9188 | ENSRNOG00000014338 | Slc25a25 | 0.255295 | 6.324152 | 20.25674 | 6.77E-06 | 0.017728 |
| 501 | ENSRNOG00000046667 | Fosb | 0.931675 | 3.991361 | 19.98715 | 7.80E-06 | 0.01786 |
| 27036 | ENSRNOG00000046968 | Nol8 | -2.17298 | 0.167337 | 19.37564 | 1.07E-05 | 0.021862 |
| 31756 | ENSRNOG00000046007 | Cldn3 | -4.12969 | -2.90796 | 18.74257 | 1.50E-05 | 0.027417 |
| 22814 | ENSRNOG00000009718 | Pex12 | -2.81063 | -0.85792 | 18.52189 | 1.68E-05 | 0.027983 |
| 21838 | ENSRNOG00000059406 | Ier5 | 0.421253 | 6.822941 | 17.93458 | 2.29E-05 | 0.034915 |
| 32597 | ENSRNOG00000029677 | AY172581.6 | -1.0246 | 1.010885 | 17.50501 | 2.87E-05 | 0.040395 |
| 12364 | ENSRNOG00000000130 | Dnajb5 | 0.313022 | 7.160973 | 17.27563 | 3.23E-05 | 0.042321 |
| 10700 | ENSRNOG00000010574 | Ptpn1 | 0.364407 | 4.985834 | 17.07803 | 3.59E-05 | 0.04383 |
| 32487 | ENSRNOG00000046544 | NA | -3.36 | -2.88431 | 16.83856 | 4.07E-05 | 0.046614 |
| 16813 | ENSRNOG00000015434 | Midn | 0.379124 | 5.949736 | 16.62973 | 4.54E-05 | 0.04716 |
| 24528 | ENSRNOG00000021731 | N4bp3 | -2.7711 | -0.92376 | 16.59302 | 4.63E-05 | 0.04716 |
| 11966 | ENSRNOG00000018484 | Plk3 | 0.494109 | 3.543355 | 16.41528 | 5.09E-05 | 0.049069 |
| 17642 | ENSRNOG00000047477 | NA | -6.76463 | -2.96655 | 16.15842 | 5.83E-05 | 0.053383 |
| 24216 | ENSRNOG00000047205 | NA | -1.98412 | -1.80855 | 15.70345 | 7.41E-05 | 0.062991 |
| 12782 | ENSRNOG00000006877 | Efnb1 | 0.410659 | 4.217455 | 15.66479 | 7.56E-05 | 0.062991 |
| 27952 | ENSRNOG00000014363 | Arhgef3 | 0.290269 | 7.257038 | 15.22897 | 9.52E-05 | 0.075879 |
| 23825 | ENSRNOG00000007387 | Per1 | 0.508601 | 6.571633 | 14.86942 | 0.000115 | 0.085711 |
| 13897 | ENSRNOG00000056135 | Tsc22d3 | 0.380317 | 6.770402 | 14.84164 | 0.000117 | 0.085711 |
| 9277 | ENSRNOG00000005600 | Nr4a2 | 0.60786 | 5.007987 | 14.61006 | 0.000132 | 0.093185 |
| 6512 | ENSRNOG00000007152 | Bhlhe40 | 0.257703 | 7.400938 | 14.44305 | 0.000144 | 0.098052 |
| 27285 | ENSRNOG00000053706 | Lonrf1 | 0.281322 | 4.938877 | 14.12089 | 0.000171 | 0.111586 |
| 24664 | ENSRNOG00000003348 | Rasd1 | 0.341275 | 4.890049 | 14.06521 | 0.000177 | 0.111586 |
| 13632 | ENSRNOG00000034240 | Bcor | 0.340279 | 5.069182 | 13.99098 | 0.000184 | 0.11221 |
| 24487 | ENSRNOG00000052660 | Gm25296 | -2.68973 | -2.21829 | 13.89486 | 0.000193 | 0.114287 |
| 9321 | ENSRNOG00000014071 | Ddb2 | -0.79847 | 0.864108 | 13.51892 | 0.000236 | 0.133145 |
| 9749 | ENSRNOG00000007478 | Cry2 | 0.212817 | 7.494252 | 13.37496 | 0.000255 | 0.133145 |
| 9854 | ENSRNOG00000017556 | Chrm4 | 0.321037 | 5.091199 | 13.37416 | 0.000255 | 0.133145 |
| 32024 | ENSRNOG00000021312 | Dnaaf5 | 0.451089 | 3.144988 | 13.36922 | 0.000256 | 0.133145 |
| 15151 | ENSRNOG00000058891 | Cys1 | 0.395549 | 3.297399 | 13.27611 | 0.000269 | 0.133145 |
| 32122 | ENSRNOG00000001379 | Cyp3a62 | -0.63087 | 1.901865 | 13.23082 | 0.000275 | 0.133145 |
| 10178 | ENSRNOG00000044661 | NA | -5.77939 | -3.18573 | 13.22607 | 0.000276 | 0.133145 |
| 209 | ENSRNOG00000036592 | Zfp518a | -0.35339 | 4.08111 | 13.14415 | 0.000288 | 0.135203 |
| 17225 | ENSRNOG00000012886 | Maff | 1.12244 | -0.08915 | 13.1012 | 0.000295 | 0.135203 |
| 28262 | ENSRNOG00000002176 | Nectin3 | -0.25912 | 4.886406 | 12.94414 | 0.000321 | 0.138275 |
| 20350 | ENSRNOG00000019107 |  | 1.31667 | -0.23983 | 12.91876 | 0.000325 | 0.138275 |
| 27905 | ENSRNOG00000023509 | Irs2 | 0.448801 | 6.844537 | 12.89973 | 0.000329 | 0.138275 |
| 20682 | ENSRNOG00000020254 | Per2 | 0.339543 | 6.654521 | 12.84593 | 0.000338 | 0.138275 |
| 5114 | ENSRNOG00000039473 | Ccdc152 | -3.20018 | -2.65847 | 12.82523 | 0.000342 | 0.138275 |
| 28924 | ENSRNOG00000030285 | Epha3 | -0.35513 | 4.103637 | 12.7975 | 0.000347 | 0.138275 |
| 22143 | ENSRNOG00000032206 | NA | -0.28611 | 5.474792 | 12.67744 | 0.00037 | 0.144305 |
| 24662 | ENSRNOG00000002997 | Slc9a3r2 | 0.29605 | 5.269922 | 12.59545 | 0.000387 | 0.145995 |
| 28258 | ENSRNOG00000059539 | NA | -0.84117 | 2.754817 | 12.57414 | 0.000391 | 0.145995 |
| 32591 | ENSRNOG00000032112 | AY172581.14 | -1.9896 | -2.32303 | 12.51575 | 0.000404 | 0.145995 |
| 15476 | ENSRNOG00000057724 | NA | 0.545619 | 3.028553 | 12.50302 | 0.000406 | 0.145995 |
| 14985 | ENSRNOG00000007329 | Frmd6 | 0.30622 | 5.764033 | 12.45164 | 0.000418 | 0.147181 |
| 15373 | ENSRNOG00000059389 | AABR07065438.2 | -0.29501 | 6.961231 | 12.26809 | 0.000461 | 0.159322 |
| 26536 | ENSRNOG00000018714 | NA | 0.298568 | 5.362994 | 12.06673 | 0.000513 | 0.174198 |
| 7148 | ENSRNOG00000025735 | Wdr86 | -3.4949 | -2.68179 | 11.94825 | 0.000547 | 0.182256 |
| 3570 | ENSRNOG00000024245 | Zkscan2 | -0.41031 | 3.481125 | 11.88268 | 0.000567 | 0.183035 |
| 20656 | ENSRNOG00000050258 | Ccnd3 | 0.274004 | 4.685393 | 11.84666 | 0.000578 | 0.183035 |
| 1522 | ENSRNOG00000014353 | Sympk | 0.253699 | 6.422805 | 11.81915 | 0.000586 | 0.183035 |
| 15059 | ENSRNOG00000008015 | Fos | 0.530788 | 5.384068 | 11.80955 | 0.000589 | 0.183035 |
| 180 | ENSRNOG00000022610 | Agbl1 | -1.26479 | -0.51859 | 11.7383 | 0.000612 | 0.184631 |
| 15835 | ENSRNOG00000043465 | Arc | 0.613418 | 8.674014 | 11.73134 | 0.000615 | 0.184631 |
| 20389 | ENSRNOG00000016361 | Plcd4 | -0.35321 | 3.965763 | 11.64369 | 0.000644 | 0.190415 |
| 510 | ENSRNOG00000014215 | Klf9 | 0.219297 | 7.185902 | 11.53043 | 0.000685 | 0.19707 |
| 22963 | ENSRNOG00000004720 | NA | -0.36502 | 3.296882 | 11.52077 | 0.000688 | 0.19707 |

**Supplementary Table 3.** Differentially Expressed Genes following RNA-Seq: Nicotine Day 6 vs Saline Day 6 (FDR < 0.05; FDR <0.2)

|  | ENSEMBLID | Symbol | logFC | logCPM | LR | PValue | FDR |
| --- | --- | --- | --- | --- | --- | --- | --- |
| 28053 | ENSRNOG00000011921 | Dusp4 | 0.485389 | 5.079757 | 27.41962 | 1.64E-07 | 0.003001 |
| 3534 | ENSRNOG00000011815 | Sgk1 | -0.6371 | 6.638982 | 25.065 | 5.54E-07 | 0.005079 |
| 22396 | ENSRNOG00000003620 | Fmo3 | -2.54195 | -1.33611 | 22.96599 | 1.65E-06 | 0.010073 |
| 10018 | ENSRNOG00000007302 | Fbn1 | -0.56736 | 3.520746 | 19.60488 | 9.52E-06 | 0.043628 |
| 6882 | ENSRNOG00000059538 | Clec2g | -0.56862 | 3.908313 | 18.43171 | 1.76E-05 | 0.064266 |
| 14132 | ENSRNOG00000002365 | Itm2a | -0.47851 | 4.40403 | 18.09267 | 2.10E-05 | 0.064266 |
| 18057 | ENSRNOG00000008151 | Plscr4 | -0.79738 | 1.934618 | 17.25389 | 3.27E-05 | 0.073835 |
| 19640 | ENSRNOG00000025895 | Cavin2 | -0.58104 | 3.485774 | 17.12404 | 3.50E-05 | 0.073835 |
| 25897 | ENSRNOG00000059549 | NA | -3.19431 | -3.27644 | 16.7945 | 4.17E-05 | 0.073835 |
| 10751 | ENSRNOG00000014034 | Olfml2a | -0.65734 | 2.984915 | 16.69403 | 4.39E-05 | 0.073835 |
| 18825 | ENSRNOG00000010944 | Hyou1 | 0.246956 | 7.662049 | 16.67685 | 4.43E-05 | 0.073835 |
| 11507 | ENSRNOG00000029427 | Grhl3 | 0.826113 | 1.091795 | 16.38779 | 5.16E-05 | 0.078827 |
| 29822 | ENSRNOG00000013851 | Spry4 | 0.426127 | 4.431514 | 16.07967 | 6.07E-05 | 0.084543 |
| 12282 | ENSRNOG00000016180 | Pdp1 | 0.414355 | 7.278601 | 15.9632 | 6.46E-05 | 0.084543 |
| 24970 | ENSRNOG00000007496 | Mtmr4 | 0.205514 | 7.452512 | 15.11353 | 0.000101 | 0.123682 |
| 32368 | ENSRNOG00000001061 | Rilpl2 | 0.256915 | 4.904231 | 14.52743 | 0.000138 | 0.158214 |
| 21838 | ENSRNOG00000059406 | Ier5 | 0.336127 | 6.822941 | 14.23805 | 0.000161 | 0.167768 |
| 2498 | ENSRNOG00000057569 | Ahnak | -0.51211 | 7.238164 | 14.19528 | 0.000165 | 0.167768 |
| 2231 | ENSRNOG00000045763 | NA | 2.589456 | -2.79683 | 14.08533 | 0.000175 | 0.168504 |
| 27952 | ENSRNOG00000014363 | Arhgef3 | 0.247065 | 7.257038 | 13.83097 | 0.0002 | 0.182977 |
| 22780 | ENSRNOG00000021719 | Slfn5 | -0.48518 | 3.115423 | 13.74232 | 0.00021 | 0.182977 |
| 7141 | ENSRNOG00000020032 | Impdh1 | 0.201667 | 5.787954 | 13.48081 | 0.000241 | 0.19287 |
| 16596 | ENSRNOG00000006789 | Ddit3 | 0.368643 | 4.154122 | 13.38367 | 0.000254 | 0.19287 |
| 30217 | ENSRNOG00000021824 | Dnajb1 | 0.281873 | 6.227162 | 13.27006 | 0.00027 | 0.19287 |
| 2606 | ENSRNOG00000027724 | Plekhf1 | -0.61386 | 2.440902 | 13.16186 | 0.000286 | 0.19287 |
| 9589 | ENSRNOG00000018294 | Hspa5 | 0.575298 | 8.717811 | 13.15805 | 0.000286 | 0.19287 |
| 5825 | ENSRNOG00000012436 | Adh6 | -3.34996 | -2.93787 | 13.12482 | 0.000291 | 0.19287 |
| 12364 | ENSRNOG00000000130 | Dnajb5 | 0.243298 | 7.160973 | 13.08805 | 0.000297 | 0.19287 |
| 21230 | ENSRNOG00000006740 | Castor1 | -0.70073 | 0.602254 | 12.95011 | 0.00032 | 0.19287 |
| 27241 | ENSRNOG00000012448 | Chrnb3 | -3.79687 | -2.96679 | 12.94682 | 0.00032 | 0.19287 |
| 31571 | ENSRNOG00000000521 | Cdkn1a | 0.488563 | 3.077199 | 12.8581 | 0.000336 | 0.19287 |
| 21898 | ENSRNOG00000002525 | Ptgs2 | 0.408309 | 6.690759 | 12.79702 | 0.000347 | 0.19287 |
| 5583 | ENSRNOG00000015691 | Inka2 | 0.177487 | 8.147043 | 12.7963 | 0.000347 | 0.19287 |
| 16129 | ENSRNOG00000004659 | Creld2 | 0.620255 | 3.481465 | 12.5638 | 0.000393 | 0.195336 |
| 28906 | ENSRNOG00000001859 | Sdf2l1 | 0.715736 | 2.850714 | 12.46532 | 0.000415 | 0.195336 |
| 31330 | ENSRNOG00000000640 | Egr2 | 0.654466 | 4.823438 | 12.43437 | 0.000422 | 0.195336 |
| 26730 | ENSRNOG00000002461 | Nid1 | -0.52194 | 3.479083 | 12.415 | 0.000426 | 0.195336 |
| 26425 | ENSRNOG00000014007 | Gfod1 | 0.297842 | 6.282167 | 12.26478 | 0.000462 | 0.195336 |
| 20876 | ENSRNOG00000003553 | Efemp1 | -0.59115 | 2.733128 | 12.21521 | 0.000474 | 0.195336 |
| 23625 | ENSRNOG00000042912 | Mycbpap | -0.32647 | 3.165592 | 12.21311 | 0.000475 | 0.195336 |
| 27010 | ENSRNOG00000018911 | Pfkfb3 | 0.176074 | 6.141017 | 12.17355 | 0.000485 | 0.195336 |
| 23138 | ENSRNOG00000002793 | Sstr2 | 0.320843 | 4.787399 | 12.10644 | 0.000502 | 0.195336 |
| 6233 | ENSRNOG00000011913 | Cp | -0.55037 | 2.920869 | 12.07196 | 0.000512 | 0.195336 |
| 30964 | ENSRNOG00000000443 | NA | -0.90117 | 0.79813 | 12.0548 | 0.000517 | 0.195336 |
| 3327 | ENSRNOG00000056228 | Atp10a | -0.51483 | 3.342447 | 12.02804 | 0.000524 | 0.195336 |
| 10021 | ENSRNOG00000047466 | Bdnf | 0.382519 | 5.515449 | 11.98439 | 0.000536 | 0.195336 |
| 7519 | ENSRNOG00000059227 | Akap3 | -0.93036 | 0.033806 | 11.98395 | 0.000537 | 0.195336 |
| 932 | ENSRNOG00000018086 | Slc22a8 | -0.68093 | 2.310891 | 11.97595 | 0.000539 | 0.195336 |
| 32534 | ENSRNOG00000000902 | Hsph1 | 0.228228 | 8.495423 | 11.96978 | 0.000541 | 0.195336 |
| 30963 | ENSRNOG00000030729 | NA | -1.09064 | -0.60406 | 11.96923 | 0.000541 | 0.195336 |
| 5457 | ENSRNOG00000011821 | S100a4 | -0.97609 | 1.063448 | 11.9598 | 0.000544 | 0.195336 |

**Supplementary Table 4.** Differentially Expressed Genes following RNA-Seq: Saline Day 6 vs Cocaine Day 6 (FDR < 0.05; FDR <0.2)

|  | ENSEMBLID | Symbol | logFC | logCPM | LR | PValue | FDR |
| --- | --- | --- | --- | --- | --- | --- | --- |
| 28053 | ENSRNOG00000011921 | Dusp4 | 0.616635 | 5.079757 | 35.7651 | 2.23E-09 | 4.08E-05 |
| 1108 | ENSRNOG00000046737 | NA | -5.98835 | -2.60486 | 32.42627 | 1.24E-08 | 0.000113 |
| 20771 | ENSRNOG00000052352 | NA | -5.30012 | -3.17197 | 23.96242 | 9.82E-07 | 0.006001 |
| 21838 | ENSRNOG00000059406 | Ier5 | 0.471243 | 6.822941 | 22.41283 | 2.20E-06 | 0.010075 |
| 31395 | ENSRNOG00000001189 | Sik1 | 0.560635 | 4.669227 | 19.24607 | 1.15E-05 | 0.042116 |
| 14132 | ENSRNOG00000002365 | Itm2a | -0.55005 | 4.40403 | 18.18421 | 2.01E-05 | 0.05231 |
| 16690 | ENSRNOG00000008415 | Nab2 | 0.399873 | 5.655164 | 18.14543 | 2.05E-05 | 0.05231 |
| 11966 | ENSRNOG00000018484 | Plk3 | 0.51455 | 3.543355 | 17.68006 | 2.61E-05 | 0.05231 |
| 19612 | ENSRNOG00000049171 | NA | 1.679456 | -0.91488 | 17.6434 | 2.66E-05 | 0.05231 |
| 12364 | ENSRNOG00000000130 | Dnajb5 | 0.313774 | 7.160973 | 17.35611 | 3.10E-05 | 0.05231 |
| 19041 | ENSRNOG00000010060 | Panx1 | 0.35796 | 5.208354 | 17.33121 | 3.14E-05 | 0.05231 |
| 23138 | ENSRNOG00000002793 | Sstr2 | 0.417647 | 4.787399 | 16.43861 | 5.03E-05 | 0.076742 |
| 31330 | ENSRNOG00000000640 | Egr2 | 0.826118 | 4.823438 | 16.12602 | 5.93E-05 | 0.083544 |
| 15835 | ENSRNOG00000043465 | Arc | 0.715549 | 8.674014 | 15.91867 | 6.61E-05 | 0.084398 |
| 30722 | ENSRNOG00000000512 | Slc26a8 | -0.89326 | 4.002298 | 15.83587 | 6.91E-05 | 0.084398 |
| 29822 | ENSRNOG00000013851 | Spry4 | 0.457193 | 4.431514 | 14.92004 | 0.000112 | 0.128471 |
| 28665 | ENSRNOG00000024127 | Atp13a5 | -0.48169 | 3.288703 | 14.33787 | 0.000153 | 0.164676 |
| 17883 | ENSRNOG00000004540 | Clec3b | -1.16571 | 0.155855 | 14.09318 | 0.000174 | 0.173104 |
| 2281 | ENSRNOG00000016926 | Plekhs1 | -1.81 | -1.70661 | 14.03468 | 0.000179 | 0.173104 |
| 17144 | ENSRNOG00000023896 | Dusp6 | 0.349183 | 6.239262 | 13.78519 | 0.000205 | 0.187791 |

**Supplementary Figure 1.** Clustered heatmap showing Pearson correlations between behavioural measures and gene expression levels for nicotine self-administration data.


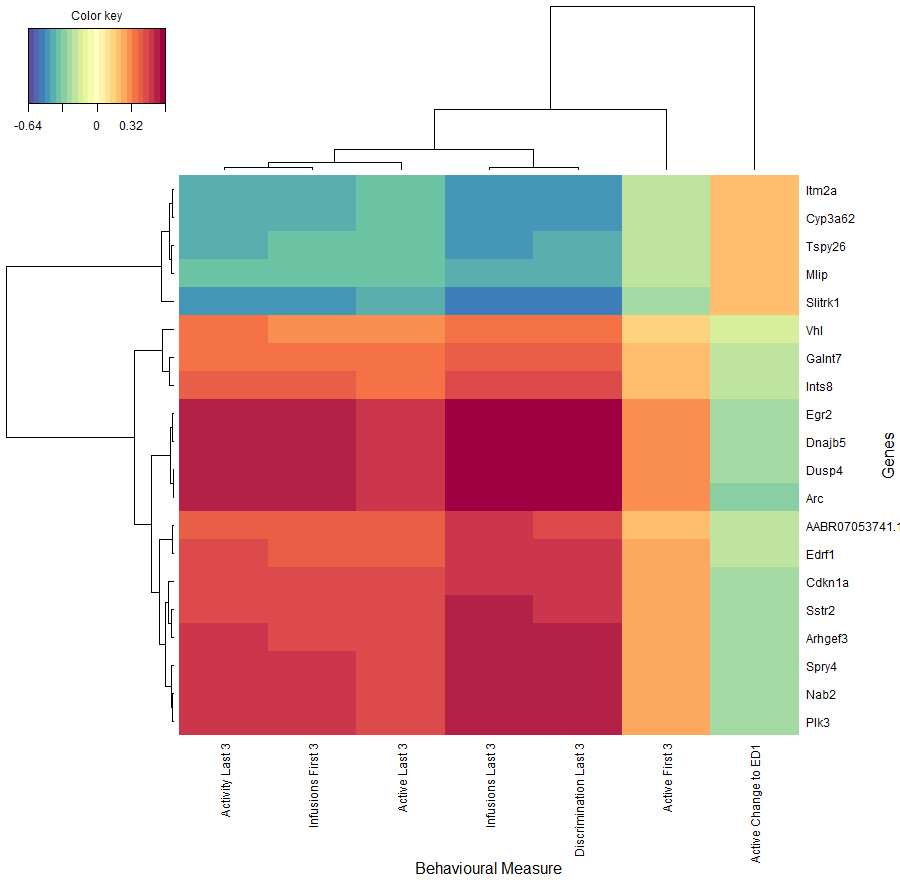

Supplement: Supplementary file 1 — Supplementary Material 1 [file 10571_2026_1667_MOESM1_ESM.docx]
